# Crystal structure of *catena*-poly[2-bromo­ethyl­ammonium [tin(II)-tri-μ-bromido]]

**DOI:** 10.1107/S2056989026006079

**Published:** 2026-06-12

**Authors:** Valeriia M. Ovdenko, Mircea-Odin Apostu, Irina A. Golenya, Anna Yu. Myronenko, Il’ya A. Gural’skiy

**Affiliations:** a Department of Chemistry, Taras Shevchenko National University of Kyiv, Volodymyrska St. 64/13, Kyiv 01601, Ukraine; b Department of Chemistry, Faculty of Chemistry, Al. I. Cuza University of Iasi, 11 Carol I Blvd, Iasi 700506, Romania; Vienna University of Technology, Austria

**Keywords:** crystal structure, hybrid perovskite like compound, tin(II), polymeric chain

## Abstract

In the structure of the title salt, {(C_2_H_7_BrN)[SnBr_3_]}_*n*_, the tin(II)atom features a strongly distorted octa­hedral environment ensured by six bromido ligands. By face-sharing, these SnBr_6_ coordination octa­hedra are connected into polymeric chains, which propagate along the *b-*axis direction. Organic cations, stabilized in a *gauche* conformation, inter­leave the inorganic polymeric chains. Hydrogen bonds of the type N—H⋯Br between organic cations and inorganic chains create supra­molecular layers parallel to the *ab* plane. These layers inter­act with each other thought weak C—H⋯Br contacts.

## Abstract 2

In the crystal structure of {(C_2_H_7_BrN)[SnBr_3_]}_*n*_, inorganic polymeric chains created by face-sharing SnBr_6_ octa­hedra are inter­leaved by organic cations of 2-bromo­ethyl­ammonium.

## 1. Chemical context

Hybrid organic–inorganic metal halides have emerged as an important class of semiconducting materials owing to their optical and electronic properties and their applicability in a wide range of optoelectronic devices, including solar cells, light-emitting diodes, photodetectors, and lasers (Zhang *et al.*, 2023 ▸). The archetypal halide perovskites adopt the general formula *ABX_3_*, where *A* is a monovalent organic or inorganic cation, *B* is a divalent metal cation (commonly Pb^2+^ or Sn^2+^), and *X* is a halide anion. In the corresponding crystal structures, corner-sharing *BX*_6_ octa­hedra form an extended framework that is responsible for their favorable charge-transport and optical properties (Li *et al.*, 2017 ▸).

Despite their outstanding performance, the toxicity of lead has stimulated intense research into alternative compositions, among which tin-based halide perovskites are considered the most promising candidates due to their suitable band gaps, strong optical absorption, and high charge-carrier mobilities (Pitaro *et al.*, 2022 ▸). However, the use of Sn^2+^-based systems can present specific challenges, including susceptibility to oxidation and a strong tendency toward structural diversity, which distinguishes them from their lead-based analogues. In particular, the stereochemically active 5*s*^2^ lone pair of Sn^2+^ often induces significant distortions of the coordination environment, leading to low-symmetry structures and a wide range of structural motifs (Stoumpos *et al.*, 2017 ▸; Sirenko *et al.*, 2024 ▸).

The periodicity of hybrid halide perovskites can be tuned by the size and functionality of the organic cations, giving rise to structures containing layers, chains, and discrete metal-halide octa­hedra (Zhou *et al.*, 2019 ▸). In such systems, the connectivity of the metal–halide octa­hedra – whether corner-, edge-, or face-sharing – plays a decisive role in determining their electronic structure and band gap. Reduced periodicity and non-corner-sharing connectivity generally lead to increased band gaps, enhanced carrier localization, and pronounced excitonic effects. Consequently, zero-, mono- and diperiodic tin halides often exhibit distinct optical properties compared to their counterparts of the *ABX*_3_ type.

Monoperiodic hybrid tin halides, in particular, have attracted growing attention due to their unique structural and physical properties. Their inorganic frameworks typically consist of chains of connected Sn–halide polyhedra, which may adopt different connectivity modes, including corner- and edge-sharing arrangements (Shi *et al.*, 2019 ▸; Spanopoulos *et al.*, 2020 ▸). These systems exhibit strong quantum confinement and enhanced electron–phonon coupling, often resulting in broadband emission that can originate from self-trapped excitons or defect-related states. Moreover, the structural flexibility of Sn^2+^ allows for the formation of highly distorted chains, which can further modulate the optical and electronic properties (Tao *et al.*, 2024 ▸).

The exploration of new tin(II) halide materials remains of considerable inter­est, particularly in relation to understanding structure–property relationships and expanding the library of perovskite-related architectures. In this context, the title compound formed unintentionally during the planned synthesis of aziridinium tin bromide (Kucheriv *et al.*, 2023 ▸) due to the high reactivity of aziridine, which can undergo ring-opening reactions in acidic media. The crystal structure of this new monoperiodic hybrid tin(II) bromide (2-BrC_2_H_4_NH_3_)[SnBr_3_] features chains constructed from bromido-bridged Sn^2+^ cations, further contributing to the structural diversity of tin halide systems.
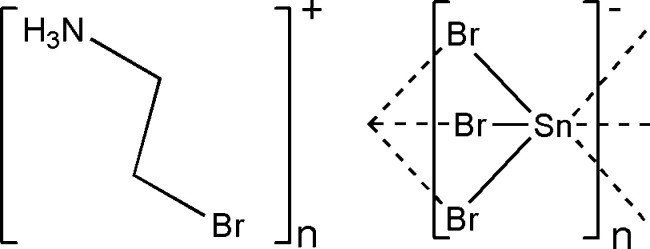


## 2. Structural commentary

**Figure 1:**
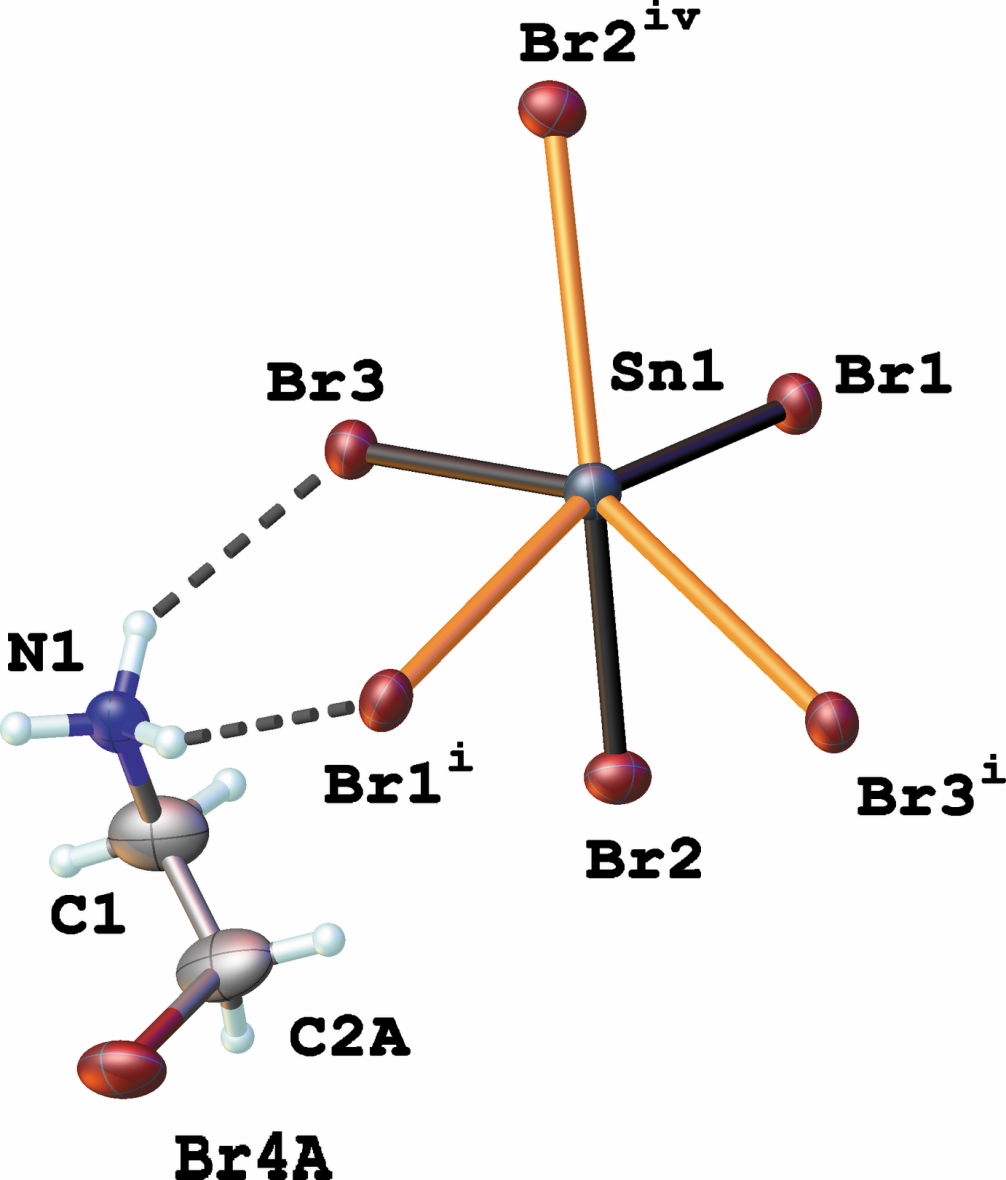
The distorted [SnBr_6_] coordination octa­hedron and the 2-bromo­ethyl­ammonium cation in the title compound. Displacement ellipsoids are drawn at the 50% probability level. Short and long Sn—Br bonds are shown in black and orange, respectively. Only one part of the disordered organic cation is given for clarity; symmetry codes refer to Table 1 ▸.

The tin(II) cation features a strongly distorted octa­hedral coordination environment provided by six bromido ligands (Fig. 1 ▸, Table 1 ▸). These inorganic octa­hedra feature three short Sn—Br bonds and three long ones, the latter being shorter than the sum of the van der Waals radii of Sn and Br (4.00 Å; Mantina *et al.*, 2009 ▸). Such a coordination environment with asymmetric trigonal distortion of the octa­hedron created by three shorter-bonded halogen anions in combination with three longer Sn—*X* contacts is quite typical for Sn^2+^ and has been observed for similar perovskite-like structures (Fabini *et al.*, 2016 ▸). The octa­hedral distortion parameters of [SnBr_6_] are very high with Δ*d* = 1/6Σ(*d_i_* − *d*)^2^/*d*^2^ = 0.0187 (where *d_i_* is one of six individual bond lengths in the octa­hedra and *d* is the mean Sn—Br bond length) and Σ = Σ|90° − α| = 165.3° (where α are the twelve *cis*-Br—Sn—Br angles). Alternatively, longer Sn—Br bonds can be considered as inter­ionic contacts between pyramidal [SnBr_3_] moieties.

**Figure 2:**
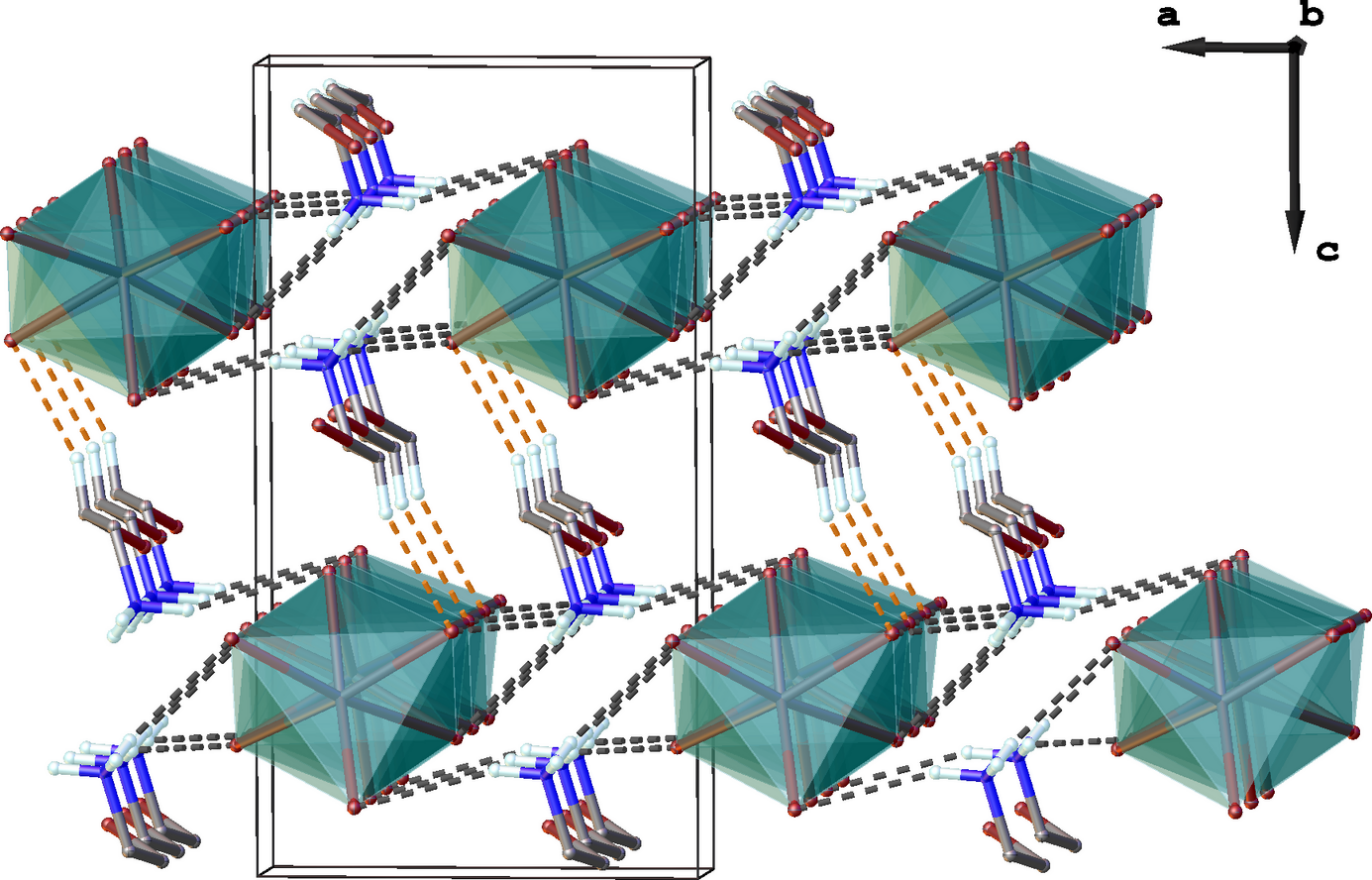
View of the inorganic chains propagating parallel to the *b* axis. N—H⋯Br inter­actions are shown as black dashed lines, C—H⋯Br bonds are shown as orange dashed lines. Disorder of the organic part and H atoms not involved in hydrogen-bonding inter­actions were omitted for clarity.

The coordination octa­hedra in this structure are further connected in a face-sharing manner into chains extending parallel to the *b* axis (Fig. 2 ▸). The negative charge of inorganic chains is balanced by organic 2-bromo­ethyl­ammonium cations, in which atoms Br4 and C2 are disordered in a nearly 1:1 ratio over two sets of sites. The observed bond lengths in the organic cation are within the expected ranges (Table 1 ▸), with the conformation being *gauche* with Br4*A*—C2*A*—C1—N1 and Br4*B*—C2*B*—C1—N1 torsion angles of 51 (2) and −58.2 (17)°, respectively.

## 3. Supra­molecular features

Inorganic chains inter­act with organic counter-ions through a set of N—H⋯·Br hydrogen bonds (Table 2 ▸, Figs. 2 ▸ and 3 ▸). Each protonated amino group creates three hydrogen bonds with bromide ions connecting two neighboring inorganic chains into supra­molecular layers parallel to the *ab* plane. These supra­molecular layers inter­act though weak C—H⋯Br inter­actions (Table 2 ▸, Fig. 2 ▸).

## 4. Database survey

A search of the Cambridge Structure Database (CSD; version 6.0, updated November 2025; Groom *et al.*, 2016 ▸) revealed four crystal structures containing tin halides in combination with 2-halogeno­ethyl­ammonium cations. These compounds were considered because chemically similar 2-halogeno­ethyl­ammonium cations often direct the structural set-up of related organic–inorganic hybrid compounds. Two of them are Sn^4+^-hybrid compounds containing discrete [Sn*X*_6_]^2–^ (*X* = Cl, Br) octa­hedra surrounded by 2-chloro­ethyl­ammonium cations (CSD refcodes KOVQOF, KOVQIZ; Elghoul *et al.*, 2024 ▸). The third compound is (2-bromo­ethyl­ammonium)_2_[SnBr_6_] (USOTAB; Kreiman *et al.*, 2026 ▸), which is isostructural to the two mentioned above, while the fourth compound is (2-iodo­ethyl­ammonium)_2_[SnI_4_], in which corner-sharing [Sn^II^I_6_]^4–^ octa­hedra create infinite layers which are inter­leaved by organic cations (TEGROQ; Song *et al.*, 2022 ▸).

The title compound differs from these previously reported structures by containing Sn^2+^ cations and a *catena*-poly[tri-μ_2_-bromido­stannate(II)] inorganic substructure extending into infinite chains.

## 5. Synthesis and crystallization

The title compound was obtained unintentionally upon the planned synthesis of (aziridinium)SnBr_3_ by the vapor diffusion method; the single-crystal X-ray diffraction experiment established the formation of (2-BrC_2_H_4_NH_3_)[SnBr_3_] instead of the target perovskite. Tin(II) chloride (150 mg, 0.79 mmol, 1 eq.) was dissolved in 1 ml of water and 0.1 ml of hydro­chloric acid (37% *w*/*w*) to avoid hydrolysis. To this solution 0.5 ml of ammonia solution (25% *w*/*w*) were added and stirred. As a result, a white precipitate of Sn(OH)_2_ was formed. The precipitate was filtered off and washed with water. The obtained tin hydroxide was dissolved in a mixture of 2.4 ml of hydro­bromic acid (48% *w*/*w*) and 4.5 ml of water. 0.2 ml of this acidic solution of tin(II) bromide was placed in a 1 ml vial. This vial was placed in a larger vial containing aziridine. The colourless crystals that formed within 1 h were collected immediately and kept in Paratone oil prior to measurements.

## 6. Refinement

Crystal data, data collection and structure refinement details are summarized in Table 3 ▸. The positional disorder of the organic cation was modelled over two set of sites, with C2 and Br4 atoms disordered over two positions and C1 and N1 atoms not being disordered. The sum of occupancies for disordered atoms was set to unity. The refined occupancies are 0.526 (18) and 0.474 (18) for parts *A* and *B*, respectively. H atoms were placed at calculated positions and refined with *U*_iso_(H) = 1.2*U*_eq_(C), *U*_iso_(H) = 1.2*U*_eq_(N). H atoms of secondary CH_2_ groups were refined as riding, while H atoms of NH_3_^+^ groups were refined as rotating. The crystal under investigation was found to be twinned by a 180° rotation around [100], and the intensity data processed into a HKLF5-type file; the twin components refined to a ratio of 0.6570 (11) : 0.3430 (11).

## Supplementary Material

Crystal structure: contains datablock(s) I. DOI: 10.1107/S2056989026006079/wm5802sup1.cif

Structure factors: contains datablock(s) I. DOI: 10.1107/S2056989026006079/wm5802Isup2.hkl

CCDC reference: 2560931

Additional supporting information:  crystallographic information; 3D view; checkCIF report

## Figures and Tables

**Figure 3:**
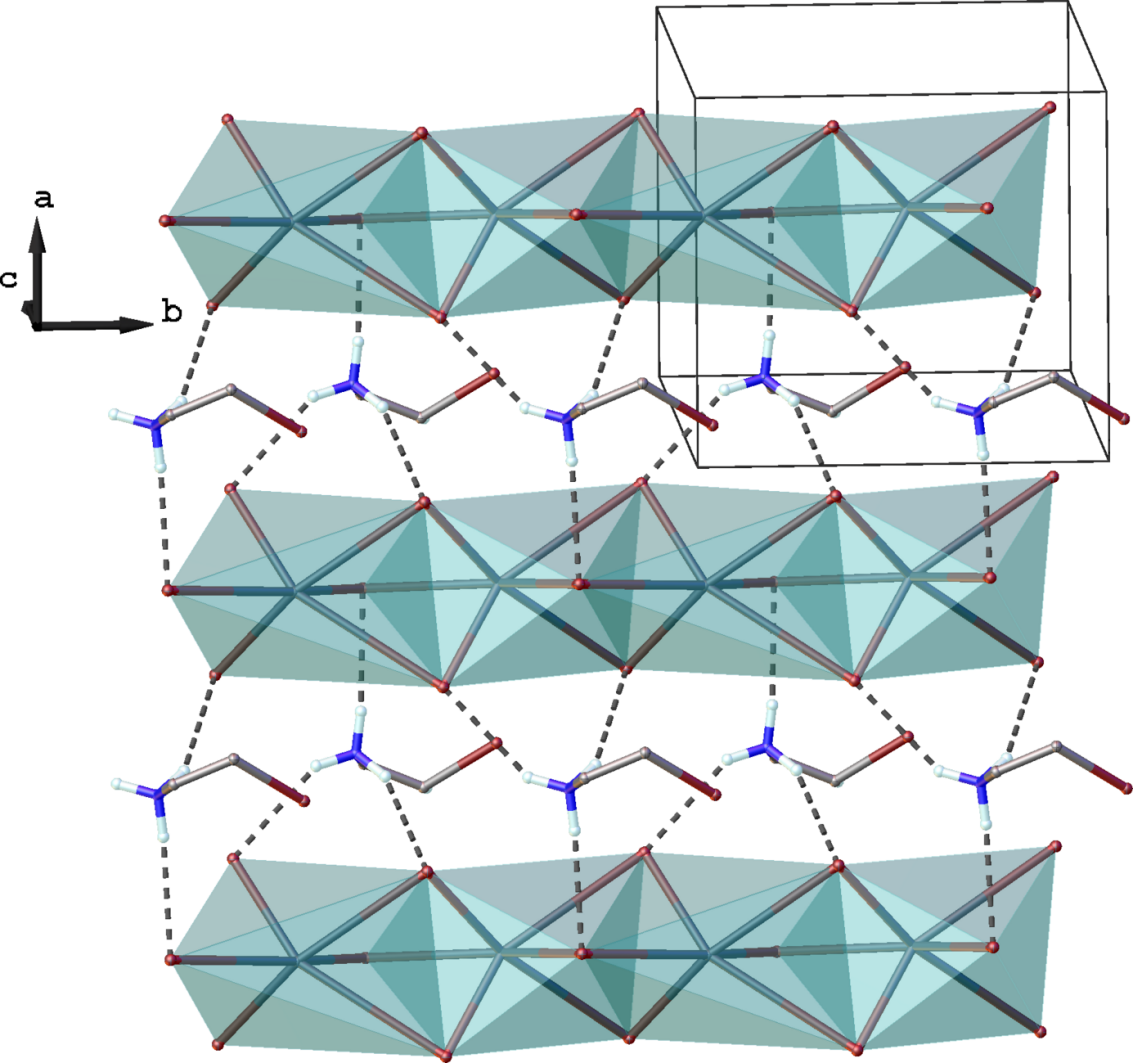
The supra­molecular layer created by means of N—H⋯Br hydrogen bonds. Disorder of the organic part and H atoms not involved in hydrogen-bonding inter­actions were omitted for clarity.

**Table 1:** Selected bond lengths (Å)

Sn1—Br1	2.7256 (10)	Br4*A*—C2*A*	1.921 (19)
Sn1—Br2	2.7228 (9)	Br4*B*—C2*B*	1.968 (19)
Sn1—Br3	2.6869 (11)	N1—C1	1.487 (11)
Sn1—Br2^i^	3.3922 (9)	C1—C2*A*	1.40 (2)
Sn1—Br1^ii^	3.4518 (10)	C1—C2*B*	1.49 (2)
Sn1—Br3^ii^	3.7621 (9)		

Symmetry codes: (i) 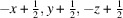; (ii) 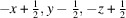.

**Table 2:** Hydrogen-bond geometry (Å, °)

*D*—H⋯*A*	*D*—H	H⋯*A*	*D*⋯*A*	*D*—H⋯*A*
N1—H1*A*⋯Br1^ii^	0.91	2.66	3.507 (7)	156
N1—H1*B*⋯Br2^iii^	0.91	2.62	3.491 (6)	160
N1—H1*C*⋯Br3	0.91	2.64	3.425 (6)	145
C1—H1*BD*⋯Br2^iv^	0.99	2.94	3.882 (9)	159
C2*A*—H2*AA*⋯Br2	0.99	2.97	3.85 (3)	149

Symmetry codes: (ii) 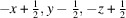; (iii) 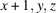; (iv) 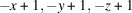.

**Table 3:** Experimental details

Crystal data	
Chemical formula	(C_2_H_7_BrN)[SnBr_3_]
*M*_r_	483.42
Crystal system, space group	Monoclinic, *P*2_1_/*n*
Temperature (K)	200
*a*, *b*, *c* (Å)	7.883 (3), 8.7065 (6), 14.4937 (11)
β (°)	90.513 (13)
*V* (Å^3^)	994.7 (4)
*Z*	4
Radiation type	Mo *K*α
μ (mm^−1^)	18.56
Crystal size (mm)	0.12 × 0.06 × 0.02

Data collection	
Diffractometer	XtaLAB Synergy, Dualflex, HyPix
Absorption correction	Analytical [*CrysAlis PRO* (Rigaku OD (2024 ▸), using a multifaceted crystal model based on expressions derived by Clark & Reid (1995 ▸)]
*T*_min_, *T*_max_	0.184, 0.648
No. of measured, independent and observed [*I* > 2σ(*I*)] reflections	3899, 3899, 3101
*R*_int_	0.034
(sin θ/λ)_max_ (Å^−1^)	0.708

Refinement	
*R*[*F*^2^ > 2σ(*F*^2^)], *wR*(*F*^2^), *S*	0.038, 0.087, 1.04
No. of reflections	3899
No. of parameters	94
H-atom treatment	H-atom parameters constrained
Δρ_max_, Δρ_min_ (e Å^−3^)	0.97, −0.69

Computer programs: *CrysAlis PRO* (Rigaku OD, 2024 ▸), *SHELXT* (Sheldrick, 2015*a* ▸), *SHELXL* (Sheldrick, 2015*b* ▸), *OLEX2* (Dolomanov *et al.*, 2009 ▸) and *publCIF* (Westrip, 2010 ▸).

## supplementary crystallographic information

### catena-Poly[2-bromoethylammonium [tin(II)-tri-µ-bromido]] Crystal data

**Table:**

(C_2_H_7_BrN)[SnBr_3_]	*F*(000) = 864
*M**_r_* = 483.42	*D*_x_ = 3.228 Mg m^−^^3^
Monoclinic, *P*2_1_/*n*	Mo *K*α radiation, λ = 0.71073 Å
*a* = 7.883 (3) Å	Cell parameters from 2192 reflections
*b* = 8.7065 (6) Å	θ = 2.7–28.4°
*c* = 14.4937 (11) Å	µ = 18.56 mm^−^^1^
β = 90.513 (13)°	*T* = 200 K
*V* = 994.7 (4) Å^3^	Plate, colourless
*Z* = 4	0.12 × 0.06 × 0.02 mm

### catena-Poly[2-bromoethylammonium [tin(II)-tri-µ-bromido]] Data collection

**Table:**

XtaLAB Synergy, Dualflex, HyPix diffractometer	3899 independent reflections
Radiation source: micro-focus sealed X-ray tube, PhotonJet (Mo) X-ray Source	3101 reflections with *I* > 2σ(*I*)
Mirror monochromator	*R*_int_ = 0.034
Detector resolution: 10.0000 pixels mm^-1^	θ_max_ = 30.2°, θ_min_ = 2.7°
ω scans	*h* = −9→9
Absorption correction: analytical [CrysAlisPro (Rigaku OD (2024), using a multifaceted crystal model based on expressions derived by Clark & Reid (1995)]	*k* = −11→11
*T*_min_ = 0.184, *T*_max_ = 0.648	*l* = −19→19
3899 measured reflections	

### catena-Poly[2-bromoethylammonium [tin(II)-tri-µ-bromido]] Refinement

**Table:**

Refinement on *F*^2^	0 restraints
Least-squares matrix: full	Hydrogen site location: inferred from neighbouring sites
*R*[*F*^2^ > 2σ(*F*^2^)] = 0.038	H-atom parameters constrained
*wR*(*F*^2^) = 0.087	*w* = 1/[σ^2^(*F*_o_^2^) + (0.0412*P*)^2^ + 2.5221*P*] where *P* = (*F*_o_^2^ + 2*F*_c_^2^)/3
*S* = 1.04	(Δ/σ)_max_ < 0.001
3899 reflections	Δρ_max_ = 0.97 e Å^−^^3^
94 parameters	Δρ_min_ = −0.69 e Å^−^^3^

### catena-Poly[2-bromoethylammonium [tin(II)-tri-µ-bromido]] Special details

**Table:**

Geometry. All esds (except the esd in the dihedral angle between two l.s. planes) are estimated using the full covariance matrix. The cell esds are taken into account individually in the estimation of esds in distances, angles and torsion angles; correlations between esds in cell parameters are only used when they are defined by crystal symmetry. An approximate (isotropic) treatment of cell esds is used for estimating esds involving l.s. planes.
Refinement. Refined as a 2-component twin.

### catena-Poly[2-bromoethylammonium [tin(II)-tri-µ-bromido]] Fractional atomic coordinates and isotropic or equivalent isotropic displacement parameters (Å^2^)

**Table:**

	*x*	*y*	*z*	*U*_iso_*/*U*_eq_	Occ. (<1)
Sn1	0.24506 (6)	0.50701 (5)	0.23958 (4)	0.02780 (14)	
Br1	0.00481 (9)	0.69436 (8)	0.31340 (6)	0.03191 (19)	
Br4A	0.7829 (14)	0.0021 (4)	0.4183 (3)	0.0494 (14)	0.526 (18)
Br4B	0.6922 (16)	−0.0059 (3)	0.4114 (2)	0.0475 (16)	0.474 (18)
Br2	0.21352 (9)	0.32682 (8)	0.39200 (6)	0.03210 (19)	
Br3	0.50363 (9)	0.65306 (8)	0.32387 (6)	0.02965 (18)	
N1	0.7777 (8)	0.3505 (7)	0.3497 (5)	0.0347 (15)	
H1A	0.723604	0.283201	0.311459	0.042*	
H1B	0.891835	0.337768	0.344926	0.042*	
H1C	0.749523	0.448271	0.333513	0.042*	
C1	0.7255 (14)	0.3215 (11)	0.4465 (7)	0.054 (2)	
H1AA	0.825780	0.333795	0.487421	0.065*	0.526 (18)
H1AB	0.641222	0.400138	0.464319	0.065*	0.526 (18)
H1BC	0.600418	0.329253	0.449890	0.065*	0.474 (18)
H1BD	0.774061	0.403046	0.486318	0.065*	0.474 (18)
C2A	0.656 (3)	0.176 (2)	0.4611 (16)	0.057 (7)	0.526 (18)
H2AA	0.542464	0.173121	0.430883	0.068*	0.526 (18)
H2AB	0.637669	0.163218	0.528206	0.068*	0.526 (18)
C2B	0.779 (3)	0.169 (2)	0.4840 (12)	0.035 (4)	0.474 (18)
H2BA	0.738155	0.159334	0.548165	0.042*	0.474 (18)
H2BB	0.904294	0.164162	0.485746	0.042*	0.474 (18)

### catena-Poly[2-bromoethylammonium [tin(II)-tri-µ-bromido]] Atomic displacement parameters (Å^2^)

**Table:**

	*U*^11^	*U*^22^	*U*^33^	*U*^12^	*U*^13^	*U*^23^
Sn1	0.0282 (3)	0.0231 (2)	0.0321 (3)	−0.00033 (18)	−0.0004 (2)	−0.0030 (2)
Br1	0.0244 (4)	0.0272 (4)	0.0441 (5)	0.0036 (3)	0.0027 (3)	0.0006 (3)
Br4A	0.065 (4)	0.0267 (10)	0.0565 (14)	0.0088 (13)	0.0095 (17)	0.0081 (8)
Br4B	0.068 (5)	0.0285 (10)	0.0456 (14)	−0.0016 (13)	0.0030 (16)	0.0022 (8)
Br2	0.0382 (4)	0.0246 (4)	0.0334 (4)	−0.0019 (3)	−0.0025 (3)	0.0024 (3)
Br3	0.0239 (3)	0.0280 (4)	0.0371 (5)	−0.0018 (3)	−0.0010 (3)	0.0008 (3)
N1	0.035 (3)	0.023 (3)	0.046 (4)	0.004 (2)	−0.002 (3)	−0.003 (3)
C1	0.082 (7)	0.038 (5)	0.042 (5)	0.009 (4)	0.015 (5)	−0.009 (4)
C2A	0.077 (16)	0.045 (11)	0.049 (13)	0.006 (10)	0.034 (12)	0.001 (10)
C2B	0.030 (10)	0.044 (10)	0.030 (9)	−0.003 (7)	−0.016 (8)	0.005 (7)

### catena-Poly[2-bromoethylammonium [tin(II)-tri-µ-bromido]] Geometric parameters (Å, º)

**Table:**

Sn1—Br1	2.7256 (10)	N1—C1	1.487 (11)
Sn1—Br2	2.7228 (9)	C1—H1AA	0.9900
Sn1—Br3	2.6869 (11)	C1—H1AB	0.9900
Sn1—Br2^i^	3.3922 (9)	C1—H1BC	0.9900
Sn1—Br1^ii^	3.4518 (10)	C1—H1BD	0.9900
Sn1—Br3^ii^	3.7621 (9)	C1—C2A	1.40 (2)
Br4A—C2A	1.921 (19)	C1—C2B	1.49 (2)
Br4B—C2B	1.968 (19)	C2A—H2AA	0.9900
N1—H1A	0.9100	C2A—H2AB	0.9900
N1—H1B	0.9100	C2B—H2BA	0.9900
N1—H1C	0.9100	C2B—H2BB	0.9900
			
Br3—Sn1—Br2	88.75 (3)	N1—C1—H1AB	108.7
Br3—Sn1—Br1	93.74 (3)	N1—C1—H1BC	108.6
Br2—Sn1—Br1	87.56 (3)	N1—C1—H1BD	108.6
Br3—Sn1—Br2^i^	77.92 (2)	N1—C1—C2B	114.5 (10)
Br2—Sn1—Br2^i^	159.89 (3)	H1AA—C1—H1AB	107.6
Br1—Sn1—Br2^i^	78.47 (3)	H1BC—C1—H1BD	107.6
Br3—Sn1—Br1^ii^	92.40 (3)	C2A—C1—N1	114.2 (11)
Br2—Sn1—Br1^ii^	77.44 (3)	C2A—C1—H1AA	108.7
Br1—Sn1—Br1^ii^	163.66 (3)	C2A—C1—H1AB	108.7
Br2^i^—Sn1—Br1^ii^	117.66 (3)	C2B—C1—H1BC	108.6
Br3—Sn1—Br3^ii^	152.91 (3)	C2B—C1—H1BD	108.6
Br2—Sn1—Br3^ii^	71.04 (2)	Br4A—C2A—H2AA	108.0
Br1—Sn1—Br3^ii^	102.93 (3)	Br4A—C2A—H2AB	108.0
Br2^i^—Sn1—Br3^ii^	125.98 (2)	C1—C2A—Br4A	117.2 (14)
Br1^ii^—Sn1—Br3^ii^	66.24 (3)	C1—C2A—H2AA	108.0
Sn1—Br1—Sn1^i^	89.10 (3)	C1—C2A—H2AB	108.0
H1A—N1—H1B	109.5	H2AA—C2A—H2AB	107.2
H1A—N1—H1C	109.5	Br4B—C2B—H2BA	108.9
H1B—N1—H1C	109.5	Br4B—C2B—H2BB	108.9
C1—N1—H1A	109.5	C1—C2B—Br4B	113.5 (11)
C1—N1—H1B	109.5	C1—C2B—H2BA	108.9
C1—N1—H1C	109.5	C1—C2B—H2BB	108.9
N1—C1—H1AA	108.7	H2BA—C2B—H2BB	107.7
			
N1—C1—C2A—Br4A	51 (2)	N1—C1—C2B—Br4B	−58.2 (17)

Symmetry codes: (i) −*x*+1/2, *y*+1/2, −*z*+1/2; (ii) −*x*+1/2, *y*−1/2, −*z*+1/2.

### catena-Poly[2-bromoethylammonium [tin(II)-tri-µ-bromido]] Hydrogen-bond geometry (Å, º)

**Table:**

*D*—H···*A*	*D*—H	H···*A*	*D*···*A*	*D*—H···*A*
N1—H1*A*···Br1^ii^	0.91	2.66	3.507 (7)	156
N1—H1*B*···Br2^iii^	0.91	2.62	3.491 (6)	160
N1—H1*C*···Br3	0.91	2.64	3.425 (6)	145
C1—H1*BD*···Br2^iv^	0.99	2.94	3.882 (9)	159
C2*A*—H2*AA*···Br2	0.99	2.97	3.85 (3)	149

Symmetry codes: (ii) −*x*+1/2, *y*−1/2, −*z*+1/2; (iii) *x*+1, *y*, *z*; (iv) −*x*+1, −*y*+1, −*z*+1.

### Sn — Br bond length (Å) in coordination octahedron.

**Table:**

	Bond length (Å)
Sn1—Br1	2.7256 (10)
Sn1—Br2	2.7228 (9)
Sn1—Br3	2.6869 (11)
Sn1—Br1^i^	3.4517 (10)
Sn1—Br2^iv^	3.3923 (9)
Sn1—Br3^i^	3.7621 (10)

Symmetry codes: (i) -x+1/2, y-1/2, -z+1/2; (iv) -x+1/2, y+1/2, -z+1/2
